# Prevalence of *BRCA1 *and *BRCA2 *mutations in unselected breast cancer patients from Greece

**DOI:** 10.1186/1897-4287-9-10

**Published:** 2011-11-15

**Authors:** Chrissovaladis Koumpis, Constantine Dimitrakakis, Aris Antsaklis, Robert Royer, Shiyu Zhang, Steven A Narod, Joanne Kotsopoulos

**Affiliations:** 1 1st Department of Obstetrics and Gynecology, Athens University Medical School, Athens, Greece; 2 Women's College Research Institute, Toronto, ON, Canada

## Abstract

**Background:**

Inheritance of a mutation in either *BRCA1 *or *BRCA2 *accounts for approximately 5% of all breast cancer cases, but varies by country. Investigations into the contribution of *BRCA *mutations to breast cancer incidence in Greece have been, for the most part, limited by small sample sizes and by the use of cases selected for their family history of cancer. The aim of the current study was to estimate *BRCA *mutation frequencies in breast cancer patients unselected for family history.

**Methods:**

To do so, we enrolled 127 unselected women with breast cancer from the Alexandra Hospital in Athens, Greece, a large public hospital in the city. Mutations in *BRCA1 *and *BRCA2 *were detected using a combination of techniques and were confirmed by direct sequencing. Two large genomic deletions were sought using mutation-specific assays. A detailed family history of cancer was obtained from each patient.

**Results:**

We were able to successfully complete testing on samples from 127 women. Among these, six mutations were identified (four in *BRCA1 *and two in *BRCA2*) representing 4.7% of the total or 9.5% of cases diagnosed before age forty. None of the mutation carriers had a family history of breast or ovarian cancer. Three of the four *BRCA1 *mutations were in exon 20: two were a G5331A mutation and the third was a 3.2 kb deletion. The fourth *BRCA1 *mutation was the 3819delGTAAA in exon 11. The two *BRCA2 *mutations were in exon 11 (3782del10 and 4512insT).

**Conclusions:**

The G5331A mutation in *BRCA1 *appears to be a founder mutation in the Greek population.

## Background

The inheritance of a deleterious mutation in one of the two breast cancer susceptibility genes, *BRCA1 *and *BRCA2*, is associated with a high lifetime risk of breast cancer, currently estimated at 80% by the age of 70 [1-3]. Deleterious mutations in both genes also increase the lifetime risks of ovarian cancer and predispose men and women to a range of other malignancies [4-6]. Factors that aid in predicting whether a mutation has been inherited by a woman with breast cancer include the number of breast and/or ovarian cancer patients in the family, the age of diagnosis, her ancestry and various pathological features of the breast cancer [7,8].

In North America, Europe, Israel and Australia, women considered to be at high-risk due to a significant family history of breast cancer may undergo molecular testing for mutations in *BRCA1 *and *BRCA2*. Genetic screening provides the opportunity for unaffected women who are identified as a carrier of a deleterious mutation to consider various risk-reducing options (e.g., prophylactic mastectomy and/or oophorectomy, tamoxifen) [9], intensive screening/surveillance (e.g., annual MRI, mammography) [10], and for women with cancer, the opportunity for individualized cancer therapy [8]. Exploring the prevalence of *BRCA *mutations in Greek patients will help establish whether the implementation of genetic services is warranted in that country.

A number of studies have evaluated the *BRCA *mutation status of breast cancer patients in Greece [11-15]. These studies have found that the common Ashkenazi Jewish mutation, 5382insC and G5331A [16], are possible founder mutations in the Greek population. Although these studies provide evidence for a role of genetics in the etiology of breast cancer in Greece, they have been limited by their small study population and the use of cases selected for a family history of cancer. Evaluation of a large Greek population of unselected cases will provide more accurate and unbiased evidence for risk associated with *BRCA *mutations and may lead to the detection of additional founder mutations. We performed mutation analysis of *BRCA1 *and *BRCA2 *on 127 unselected patients with breast cancer in Athens, Greece.

## Methods

### Patient Population

We conducted a study of unselected breast cancer patients, diagnosed at or before age 80, in Athens, Greece from February 10, 2006 to April 26, 2008. Patients were recruited from a single large public hospital in Athens (Alexandra Hospital). The study population consisted of patients undergoing treatment for a recent diagnosis of breast cancer. Unselected, newly diagnosed breast cancer cases were identified from the breast cancer clinic at Alexandra Hospital. Cases were approached about the study during their visit to the hospital. The study was introduced and described to the patient by the breast surgeon or research assistants. If the patient agreed to participate, she was provided a written description of the study, including a consent form. The patient was informed of the implications of genetic testing. At the time of the clinic appointment, a risk factor questionnaire was completed and a family history was recorded. A blood sample was obtained for DNA extraction. Mutation analysis took place in the laboratory of Dr. Steven Narod (Toronto, Canada). The institutional review boards at the participating centers approved the protocol and all of the women in the study provided written informed consent.

Patients were identified through the pathology and surgical records of the breast cancer service and were invited to participate through a special invitation. Of these, 50 were above the age of 80 and were ineligible. In total, 127 women were included in this study. On average, 3.7 years had elapsed between the date of diagnosis and the date of interview. All participants were interviewed in person for their family history of cancer, with specific reference to a history of breast or ovarian cancer. Tumor histology, tumor size, lymph node involvement and grade were abstracted from the medical records. Questionnaires on family histories and lifestyle factors were completed for all 127 cases.

All samples were screened for six common alterations, and two large genomic deletions, based on previous literature [11-17]. The common *BRCA1 *5382insC mutation was detected using a multiplex PCR reaction as previously described [17]; however, two separate screening assays were designed using restriction enzymes for two other *BRCA1 *mutations G5331A and C5370CT. All mutations were confirmed through direct sequencing. The G5586A mutation, which leads to exon 23 skipping [13], was detected by direct sequencing on all samples, and previously designed TaqMan Copy Number Variation Assays (ABI) were used for the large deletions del3.2kb, and del4429ins5 both in *BRCA1*. Assay ID numbers are available upon request. Protein truncation testing (PTT) was used to detect *BRCA2 *mutations 2024del5 and 4147delG in exons 10 and 11, respectively. PTT was performed using the TNT™ rabbit reticulocyte lysate system (Promega), involving [35S]methionine/cysteine (New England Nuclear) for protein detection. Aberrant and mutation positive bands were sequenced and confirmed by direct sequencing.

## Results

A total of 127 breast cancer patients were tested for *BRCA1 *and *BRCA2 *mutations using a combination of laboratory techniques. The mean age of the patients at diagnosis was 50.8 years (range 19.3-78.2 years) and the average age at interview was 54.4 years (range 24.1-78.5). Sixteen percent of the patients were diagnosed before the age of 40 (n = 21) and 49% were diagnosed before the age of 50 (n = 62). Seventeen (13%) of the patients had a family history of breast or ovarian cancer and 13 (10%) of the patients had first-degree relative diagnosed with breast or ovarian cancer.

Overall, six mutations were identified (4.7%; 95%CI 0.018-0.10), including four in *BRCA1 *(3.1%) and two in *BRCA2 *(1.6%) (Table 1). Three of the four *BRCA1 *mutations were in exon 20: two were the G5331A mutation and the third was the 3.2 kb deletion. The fourth *BRCA1 *mutation was 3819delGTAAA in exon11. Both the *BRCA2 *mutations were in exon 11 (3782del10 and 4512insT). A mutation was seen in two of 21 patients diagnosed with breast cancer before age 40 (9.5%), in two of 46 patients diagnosed between ages of 40 and 50 (4.3%) and in two of 60 cases diagnosed above age 50 (3.3%). One of the six mutation carriers had a family history of breast or ovarian cancer and a first-degree relative diagnosed with breast or ovarian cancer (16.7%).

**Table 1:** *BRCA1 *and *BRCA2 *Mutations Found in Greek Breast Cancer Patients

Patient	Gene	Exon	Mutation	Age at diagnosis	Family history of breast or ovarian cancer
31617	*BRCA1*	20	3.2 kb deletion	59.05	Yes
31638	*BRCA1*	20	G5331A	46.12	None
31652	*BRCA1*	11	3819delGTAAA	40.96	None
31669	*BRCA1*	20	G5331A	23.03	None
33319	*BRCA2*	11	4512insT	38.50	None
33328	*BRCA2*	11	3782del10	56.22	None

## Discussion

The aim of the current study was to estimate the prevalence of *BRCA *mutations in a series of unselected female breast cancer cases from Greece. We identified a deleterious *BRCA1 *or *BRCA2 *mutation in 4.7% of the women with breast cancer. The most common mutation was the G5331A mutation, accounting for 1.6% of the cancers diagnosed and 33% of all mutations. Four additional mutations were identified; two in *BRCA1 *and two in *BRCA2*. Other studies of unselected populations have reported relatively low *BRCA *mutation frequencies (range: *BRCA1 *0-7%; *BRCA2 *1-3%) (reviewed in [18]).

One other hospital-based study has evaluated the frequency of *BRCA1 *(but not *BRCA2*) mutations among unselected breast cancer cases in Greece [19]. The authors evaluated the prevalence of four specific *BRCA1 *mutations. They previously reported that these account for 54% of all mutations detected in both genes [19]. They found that 26 of the 987 patients (2.6%) carried one of four mutations in *BRCA1*: 5382insC (1.3%), 5331G>A (0.4%), and two large genomic rearrangements involving deletions of exons 20 (0.3%) and 24 (0.6%). 5382insC has been previously described in numerous populations while the latter three mutations have only been described in Greek populations. Further, 14 of the 26 (54%) carriers had an early age of diagnosis (<40 years) and 10% of all the early-onset patients carried one of the four mutations. Given that the original data was based on the testing of 287 Greek families with a history of breast and/or ovarian cancer, this was not a study of unselected patients.

Nearly all the other prior studies evaluating *BRCA *mutation frequencies among patients in Greece have included women with familial breast and/or ovarian cancer (i.e., those with a family history) while the women in the current study were unselected for a personal or family history of cancer [11-16]. In the most comprehensive of these studies, the authors identified 26 *BRCA *mutations (15 *BRCA1 *and 11 *BRCA2*) in 287 Greek breast/ovarian cancer families using dHPLC followed by direct sequencing [16]. Four *BRCA1 *mutations accounted for more than half of the identified mutations, while the remaining 22 mutations were a mixture of unique mutations occurring at a low frequency suggesting a genetically heterogenous population [20]. The 5382insC and G5331A mutations in exon 20 of *BRCA1 *accounted for 46% of the families. We did not detect the 5382insC frameshift mutation in our study population. Although the 5382insC mutation is known as a founder mutation in the Ashkenazi Jewish population, Hamel *et al*. recently estimated that this mutation likely arose approximately 1,800 years ago in Scandinavia or northern Russia and infiltrated the Ashkenazi Jewish population 400-500 years ago [21]. Further, the authors concluded that this is likely the most common mutation in many European countries.

Relatively few *BRCA *mutations account for *BRCA*-associated cancers among homogenous populations such as Iceland and Poland, while more heterogeneous populations such as in Canada or the United States tend to display a broad mutation spectrum [18,22,23]. Founder mutations are specific mutations that appear repeatedly in ethnically defined groups because of a shared common ancestry. Such mutations have been identified in Icelandic [24], Polish [25], French-Canadian [26], and Ashkenazi Jewish [27], among other, populations. If genetic testing is to be offered to high-risk individuals, it is more economical to screen for founder mutations rather than comprehensive genetic testing which is considerably more costly.

Based on our and prior findings [14,16,28], it appears that G5331A mutation in *BRCA1 *may be a founder mutation in the Greek population. Previous studies indicate that the 5382insC frameshift [11,13,14,16,21] is likely a founder mutation as well, although this was not the case in the current study. In addition to these two mutations, many others reported several novel mutations, deletions or genomic rearrangements as well as unclassified variants and [11,12,15,16,19] suggesting a genetically heterogeneous population.

A major strength of the current study were the inclusion of unselected breast cancer cases thus allowing for an accurate estimation of the proportion of cases due to *BRCA *mutations. Further, genetic testing for *BRCA1 *and *BRCA2 *is highly sensitive [29]. Nonetheless, we were limited by the relatively small sample size (n = 127). The majority of the aforementioned studies were limited by the inclusion of familial cases, the relatively small sample sizes (n range 25-287) and the screening of one rather than both *BRCA *genes.

## Conclusions

Inheritance of a deleterious *BRCA *mutation is one of the most important predictors of an individual's risk of developing breast cancer. Given that breast cancer is the most commonly diagnosed malignancy among women in Greece, it is of value to identify those at a high-risk of developing the disease. Genetic screening provides the opportunity for women identified as carriers of a deleterious mutation to consider various prevention strategies (e.g., prophylactic mastectomy and/or oophorectomy, tamoxifen) [9] or intensive screening (e.g., annual MRI, mammography) [10] to help lessen the burden of this disease.

## List of Abbreviations

*BRCA1*: breast cancer susceptibility gene 1; *BRCA2*: breast cancer susceptibility gene 1.

## Competing interests

The authors declare that they have no competing interests.

## Authors' contributions

SAN and JK conceived the study. CD and AA participated in the design of the study and in the supervision of the accrual of study participants. CK interviewed the participants and collected information. RR and SZ conducted the mutation analysis. All authors read and approved the final manuscript.
